# False positive ^18^FDG PET-CT results due to exogenous lipoid pneumonia secondary to oily drug inhalation: A case report: Erratum

**DOI:** 10.1097/MD.0000000000007315

**Published:** 2017-06-16

In the article, “False positive ^18^FDG PET-CT results due to exogenous lipoid pneumonia secondary to oily drug inhalation: A case report”,^[1]^ which appeared in Volume 96, Issue 22 of *Medicine,* Dr. Caroline Grangeaon's name should have appeared as Caroline Grangeon-Chapon.
